# Endothelial Retargeting of AAV9 In Vivo

**DOI:** 10.1002/advs.202103867

**Published:** 2022-01-12

**Authors:** Tarik Bozoglu, Seungmin Lee, Tilman Ziegler, Victoria Jurisch, Sanne Maas, Andrea Baehr, Rabea Hinkel, Amelie Hoenig, Anjana Hariharan, Christina Inyeop Kim, Simon Decker, Haider Sami, Tobias Koppara, Ruppert Oellinger, Oliver J. Müller, Derk Frank, Remco Megens, Peter Nelson, Christian Weber, Angelika Schnieke, Markus Sperandio, Gianluca Santamaria, Roland Rad, Alessandra Moretti, Karl‐Ludwig Laugwitz, Oliver Soehnlein, Manfred Ogris, Christian Kupatt

**Affiliations:** ^1^ Klinik und Poliklinik für Innere Medizin I University Clinic rechts der Isar Technical University Munich Germany; ^2^ DZHK (German Center for Cardiovascular Research) Partner Site Munich Heart Alliance Munich Germany; ^3^ Institute for Cardiovascular Prevention Ludwigs‐Maximilians‐University Munich Germany; ^4^ Deutsches Primatenzentrum GmbH Leibnitz‐Institut für Primatenforschung Department of Laboratory Animal Science Göttingen Germany; ^5^ Faculty of Life Sciences Department of Pharmaceutical Sciences Laboratory of MacroMolecular Cancer Therapeutics (MMCT) University of Vienna Althanstrasse 14 Vienna 1090 Austria; ^6^ Institute of Molecular Oncology and Functional Genomics University Clinic rechts der Isar Technical University of Munic Germany; ^7^ Klinik für Innere Medizin III and DZHK (German Center for Cardiovascular Research), partner site Hamburg, Kiel, Lübeck. UKSH Kiel Germany; ^8^ Medizinische Klinik und Poliklinik IV Poliklinik Innenstadt Ludwigs‐Maximilians‐University Munich Germany; ^9^ Department of Animal Sciences Chair of Livestock Biotechnology School of Life Sciences Weihenstephan Technical University Munich Germany; ^10^ Walter Brendel Centre of Experimental Medicine and Institute of Cardiovascular Physiology and Pathophysiology BioMedical Centre Ludwig Maximilians University of Munich Germany; ^11^ Institute for Experimental Pathology (ExPat) Center for Molecular Biology of Inflammation (ZMBE) WWU Münster Germany

**Keywords:** endothelium, subject terms: gene therapy, vascular biology

## Abstract

Adeno‐associated viruses (AAVs) are frequently used for gene transfer and gene editing in vivo, except for endothelial cells, which are remarkably resistant to unmodified AAV‐transduction. AAVs are retargeted here toward endothelial cells by coating with second‐generation polyamidoamine dendrimers (G2) linked to endothelial‐affine peptides (CNN). G2^CNN^ AAV9‐Cre (encoding Cre recombinase) are injected into mTmG‐mice or mTmG‐pigs, cell‐specifically converting red to green fluorescence upon Cre‐activity. Three endothelial‐specific functions are assessed: in vivo quantification of adherent leukocytes after systemic injection of ‐ G2^CNN^ AAV9 encoding 1) an artificial adhesion molecule (S1FG) in wildtype mice (day 10) or 2) anti‐inflammatory Annexin A1 (Anxa1) in ApoE^−/−^ mice (day 28). Moreover, 3) in Cas9‐transgenic mice, blood pressure is monitored till day 56 after systemic application of G2^CNN^ AAV9‐gRNAs, targeting exons 6–10 of endothelial nitric oxide synthase (eNOS), a vasodilatory enzyme. G2^CNN^ AAV9‐Cre transduces microvascular endothelial cells in mTmG‐mice or mTmG‐pigs. Functionally, G2^CNN^ AAV9‐S1FG mediates S1FG‐leukocyte adhesion, whereas G2^CNN^ AAV9‐Anxa1‐application reduces long‐term leukocyte recruitment. Moreover, blood pressure increases in Cas9‐expressing mice subjected to G2^CNN^ AAV9‐gRNA^eNOS^. Therefore, G2^CNN^ AAV9 may enable gene transfer in vascular and atherosclerosis models.

## Graphical abstract

A combination of polyamidoamine nanoparticle of second generation (G2) coating and endothelial‐affine peptide targeting is applied to alter the tropism of AAV9 from myo‐ and liver‐tropism to endothelial cells. Successful retargeting of G2^CNN^‐coated AAV9 via fluorescence microscopy and three functional assays depending on endothelial transduction of the transgene is demonstrated. This retargeting renders AAV9 utilizable for vascular investigations.

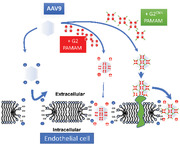

## 1 Introduction

Adeno‐associated viruses (AAVs) are favored vectors for preclinical and clinical application, harboring a broad array of supplementary,^[^ ^1^ ^]^ inhibitory,^[^ ^2^ ^]^ and gene editing transgenes.^[^ ^3^ ^]^ Parenchymal cells of a variety of organs,^[^ ^4^ ^],^ e.g., liver,^[^ ^5^ ^]^ lung,^[^ ^6^ ^]^ muscle,^[^ ^7^ ^]^ and heart,^[^ ^8^ ^]^ have been targeted by natural tropism of AAV‐serotypes. Myotropic AAVs are frequently used to target the heart in experimental (e.g., refs. [8, 9]) and clinical^[^ ^10^ ^]^ settings, with serotypes 8 or 9 favored for gene editing in translational animal models.^[^ ^3^, ^11^ ^]^ In contrast, little use has been made of AAVs in of vascular gene therapy, mostly due to a lack of transduction efficacy of vascular cells.

Several approaches to retarget AAVs toward endothelial cells were reported for AAV2 ^[^ ^12^ ^]^ and AAV9.^[^ ^13^ ^]^ Glycation of the heparan sulfate proteoglycan (HSPG) binding site of AAV6 and linkage of a single chain monoclonal antibody against vascular cell adhesion protein 1 (VCAM‐1) via click‐chemistry enhanced transduction of inflamed endothelium in vitro.^[^ ^14^ ^]^ Moreover, endothelial targeting peptides, selected from libraries, and directly expressed on the virus capsid at the amino‐acid residues R588 (AAV2) ^[^ ^12^ ^]^ or A589 (AAV9),^[^ ^13^ ^]^ disrupted the natural HSPG binding site of the AAV capsids. These peptide insertions eventually demonstrated transduction of endothelial cells of large vessels (aorta, vena cava) in vivo but not in the microvascular compartment. Although other peptide variants (PPS,^[^ ^15^ ^]^ BR1^[^ ^16^ ^]^) were successfully applied for targeting brain microvascular endothelial cells in vivo, they lacked expression in other vascular beds, e.g., heart or skeletal muscle.

**Figure 1:**
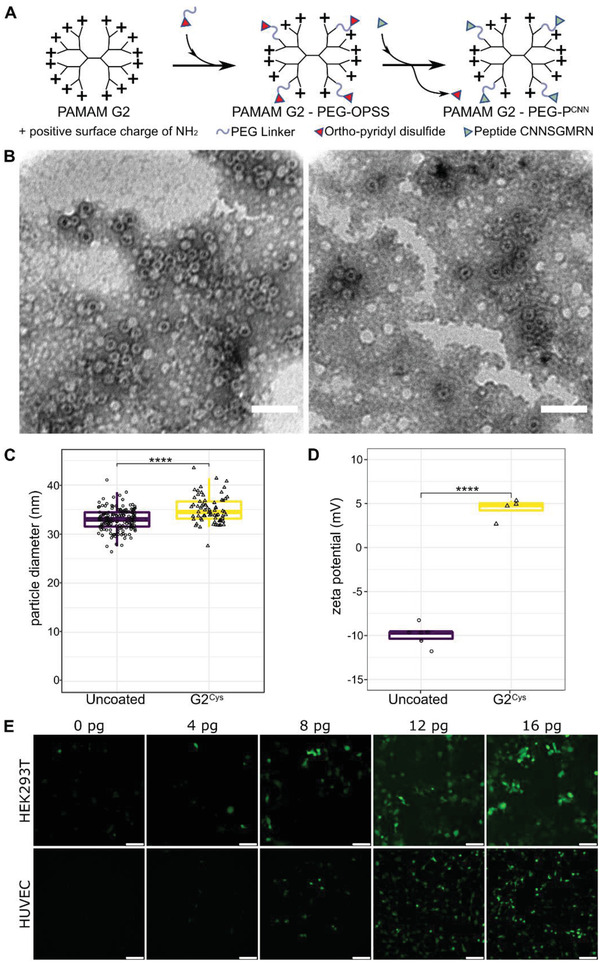
Coating of rAAV2/9 with PAMAM dendrimers. A) Generation 2 polyamidoamine dendrimers modified stepwise by PEGylation with NHS‐PEG_2K_‐OPSS and conjugated to peptides via the *N* terminal cysteine residue. B) Representative transmission electron micrograph of AAV9 (left) or AAV9 coated with PEGylated G2 PAMAM (right). (Scale bars: 100 nm). C) Comparison of particle diameters of uncoated (*N* = 153) and coated rAAV (*N* = 69, *p* < 0.0001). D) Zeta potential of uncoated or G2^Cys^ coated AAV9 particles. E) Transduction efficacy of AAV9‐GFP on a nonendothelial (Human embryonic kidney = HEK293T cells) and an endothelial cell line (human umbilical vein endothelial cells = HUVECs) at MOI of 1 × 10^6^ coated by incrementally increasing doses (pg/cell) of G2^Cys^ PAMAM (green: GFP, Scale bars: 100 µm).

Our alternate approach to retarget AAV9 from muscle toward microvascular endothelium leaves the native tropism ^[^ ^17^ ^]^ intact, but attaches the endothelial‐affine peptide (EP) to the virus capsid. For this purpose, coating with amine‐terminated, second generation polyamidoamine (PAMAM)‐dendrimers (G2, **Figure** **1**A) was used, which serve as binding matrix for the linker (ortho‐pyridyl‐2‐disulfid (**O**PSS)‐omega‐carboxy succinimidyl ester (NHS) polyethylene glycol (PEG)=OPN) carrying the EP (Figure 1A). In the current study, we tested whether coating of AAV9 capsids with PEGylated G2‐conjugates linked to EPs would suffice to allow for functionally relevant endothelial targeting of AAV9 in murine and porcine models.

## 2 Results

### 2.1 G2^CYS^‐Coating of AAV9 Facilitates Target Cell Entry without Changing Tropism

PEGylated G2‐ conjugates (G2^CYS^) readily bound to negatively charged surfaces of AAV9 capsids (Figure 1A) and significantly increased their size, as evident by the particle diameters estimated from the respective transmission electron microscopy (TEM) image (32.97 ± 2.24 nm, *n* = 153 for naked AAV9 capsids vs 35.01 nm ± 2.74, *n* = 69 for capsids coated with 450 ng G2^CYS^ per 2.5 × 10^12^ virus genomes = vgs, *p* < 0.00001) (Figure 1B,C). G2^CYS^ conjugates coating altered the zeta potential from negative to positive surface charge values (−9.9 ± 1.18 mV for AAV9 vs 4.4 ± 1.19 mV G2^CYS^ AAV9, *p* < 0.00001) (Figure 1D). Of note, this positively charged surface of the AAV9 sufficed to dose‐dependently increase AAV9‐transduction of human endothelial as well as nonendothelial cells (HUVEC and HEK293T cells, respectively) in vitro (Figure 1E).

**Figure 2:**
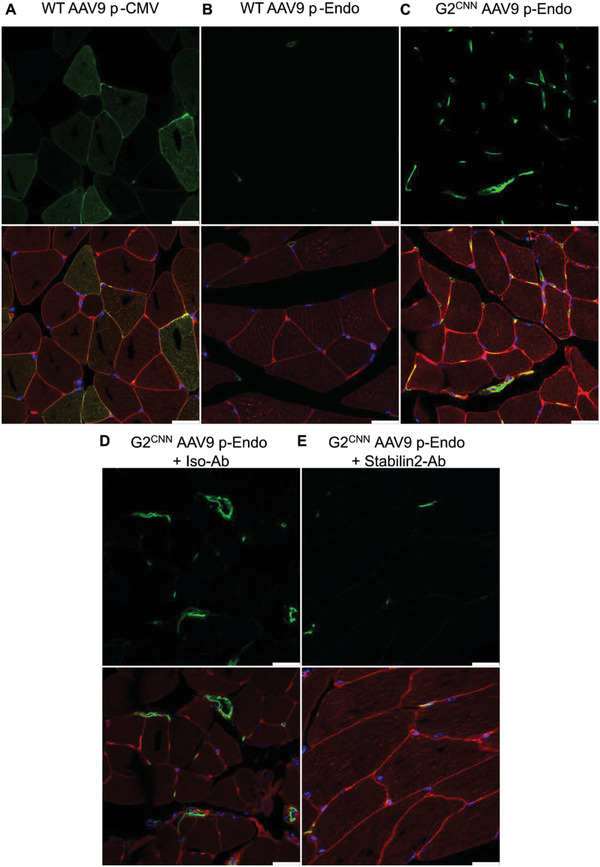
Modulation of in vivo AAV9‐Cre transduction by G2^CNN^ coating in mTmG mice. Examples of hindlimb skeletal muscle transduction of mTmG mice expressing membrane dTomato (red) unless Cre recombinase activity enables expression of enhanced green fluorescence protein (EGFP, green). Cre was transduced by A) an unmodified rAAV2/9 vector with CMV promoter, B) unmodified AAV9 pCMV Cre, or C) G2^CNN^ coated AAV9 pEndo. Cre. Transduction with D) G2^CNN^ coated AAV9 with endoglin promoter was also carried out 30 min after preinjection with isotype control IgG_k_ or E) anti‐*Stab2* antibody. Top: EGFP alone, bottom: merge of EGFP, dTomato and DAPI (blue). Scale bars represent 25 µm.

Subsequently, we investigated the effect of G2^CYS^‐coating on AAV9‐Cre transduction efficacy detected as red‐to‐green switch in mTmG‐mice, containing a floxed tdTomato‐transgene and a floxed stop‐codon in front of an EGFP (enhanced green fluorescent protein) transgene in the Rosa26 locus.^[^ ^18^ ^]^ Excision of both floxed alleles via Cre‐nuclease would provide a red‐to‐green fluorescence switch in vivo. Notably, upon systemic application of 2.5 × 10^12^ vgs, we readily obtained a skeletal myocyte (Figure S1A, Supporting Information) and cardiomyocyte (Figure S1B, Supporting Information) transduction rate of 47.3% ± 3.6% and 51.3% ± 5.9%, respectively, confirming the myotropism of AAV9. However, no clear‐cut endothelial AAV9‐Cre transduction was detectable, being potentially masked by fluorescence of neighboring cardiomyocytes. Thus, we switched from utilizing a permissive CMV‐promoter (cytomegalovirus, pCMV), to an Endoglin promoter (pEndo), which restricted Cre‐expression to endothelial cells. Removing muscle transduction by using AAV9.pEndo.Cre, we obtained only minimal endothelial AAV9‐transduction in skeletal muscle (**Figure** **2**B) and heart (Figure S1C, Supporting Information), without or with G2^CYS^‐coating.

### 2.2 G2^CNN^ Coating of AAV9‐Cre Enables Expression of a Stop‐Loxed EGFP In Vitro and In Vivo

In order to overcome the tropism stability of G2^CYS^, we attached an EP to it. After three rounds of biopanning in cultured endothelial cells using an M13‐CX7C phage library (Figure S1D, Supporting Information), we obtained a CNNSGMRN peptide (CNN), which we linked to the G2‐PAMAM via OPNs (G2^CNN^), again increasing the size compared to native AAV9 (Figure S1E, Supporting Information), similar to G2^Cys^ (Figure 1B,C). After systemic injection of G2^CNN^ AAV9 carrying p.Endo.Cre into mTmG mice, we observed a profound increase of microvascular endothelial transduction of skeletal muscle (Figure 2C) and heart (Figure S1F, Supporting Information). The Cre‐meditated red‐to‐green fluorescence switch was depending on EP^CNN^, since an antibody against the hyaluronan receptor for endocytosis (*Stab2*) , which is in part homologous to the CNN‐motif (Figure S1D, Supporting Information), abrogated endothelial transduction (Figure 2D,E and Figure S1F, Supporting Information). Quantification of Cre‐transduced and PECAM‐1 (platelet and endothelial cell adhesion molecule 1, CD31) positive endothelial cells amounted to 32.9% ± 4.1% in skeletal muscle and 42.7% ± 2.1% in the heart. These results were corroborated by flow cytometry of PECAM‐1 positive EGFP‐expressing cells after cell isolation from whole hearts. Here, addition of G2^CNN^ increased the transduction of PECAM‐1‐positive endothelial cells from 5.1% ± 2% to 27.3% ± 7.2% (Figure S2A, Supporting Information) in mice.

In order to investigate whether the high selectivity of the CNN motif itself or the molecular composition of the G2‐OPN sandwich linked to an endothelial‐affine peptide was essential for the observed endothelial targeting, we used an alternate EP, SLRSPPS ( = SLR). This peptide has been described to transduce large vessels, when expressed directly at amino acid residue A589 of the AAV9 capsid,^[^ ^13^ ^]^ without, however, providing microvascular transduction (Figure S3A, Supporting Information). Linked to G2, SLR was similarly enhancing AAV9.pEndo.Cre transduction of microvascular endothelial cells in muscle tissue, unless an antibody prevented binding of the G2^SLR^ AAV9 to its cognate receptor FGFR3 (Figure S3B, Supporting Information).

**Figure 3:**
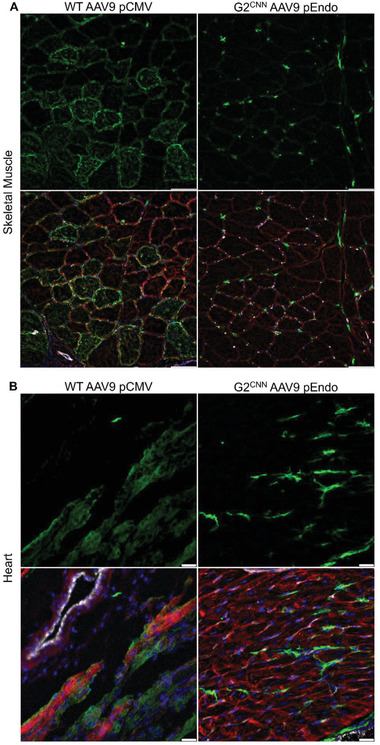
Endothelial transduction of mTmG pigs with AAV9‐Cre. mT mG^−1^ reporter pigs were transduced with either unmodified AAV9.pCMV.Cre (left) or G2^CNN^ coated AAV9.pEndo.Cre (right) via local application into A) hindlimb muscle and B) left ventricle. Top: EGFP alone (green), bottom: merge of EGFP, dTomato (red), CD31 (white), and DAPI (blue). Scale bars represent A) 100 µm or B) 25 µm.

Next, we assessed whether G2^CNN^ AAV9 encoding for pEndo.Cre would also be able to switch endothelial cells from default fluorescent red (tdTomato) to fluorescent green (EGFP), when locally applied into mTmG pigs, containing the floxed tdTomato allele together with a floxed stop‐codon in front of EGFP in the porcine Rosa26 locus.^[^ ^19^ ^]^ In contrast to AAV9.pCMV.Cre, which was incapable of activating EGFP‐expression in porcine cardiac or skeletal muscle endothelium, G2^CNN^ AAV9.pEndo.Cre readily transduced microvascular endothelia (PECAM‐1^+^) of both peripheral and heart tissue (**Figure** **3**). Local injection of the G2^CNN^ coated pEndo.Cre carrying vector sufficed to transduce 15.8% ± 0.9% of m. quadriceps and 32.7% ± 2.8% of heart muscle capillary endothelia, whereas an equivalent dose of wildtype AAV9 did not induce this effect. This observation was upheld by flow cytometric analysis of PECAM‐1 positive EGFP‐expressing cells in digested hindlimb muscle samples (10.9% ± 3.4% vs 1.8% ± 0.4%, *p* < 0.05) (Figure S2B, Supporting Information).

No obvious toxicity was obtained while systemically applying AAV9 with the G2^CNN^ modification (Figure S2C and Tables S1 and S2, Supporting Information).

### 2.3 AAV‐Mediated Expression of S1FG, an Artificial Adhesion Molecule, for Increased Leukocyte Adhesion

**Figure 4:**
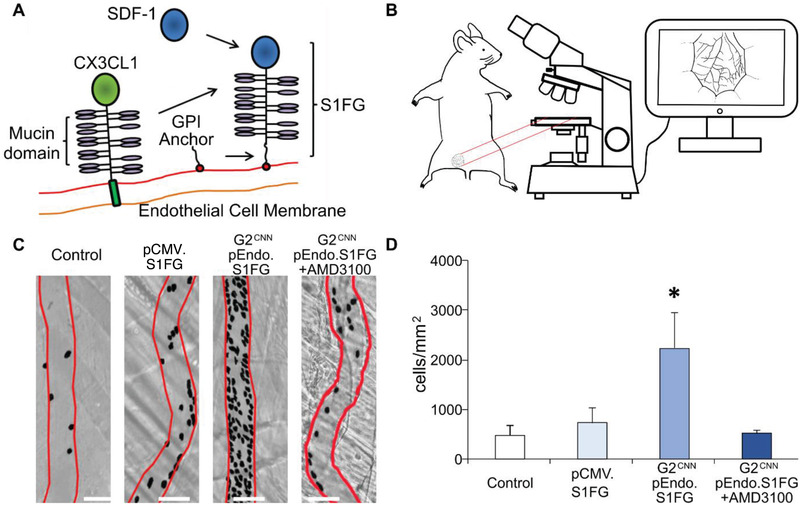
Endothelial S1FG delivery via endothelial retargeted AAV9. A) AAV9 encoding S1FG, an artificial leukocyte adhesion molecule consisting of SDF‐1, the mucin domain of CX3CL1, and a GPI‐anchor, was injected to tail veins of wild type mice. B) Intravital microscopy of cremaster was carried out. C) Muscle venules (red outline) revealed enhanced adhesion of leukocytes (black dots) upon S1FG expression. D) Adhesion of leukocytes showed a significant increase with endothelial retargeting (G2^CNN^ coated AAV9.pEndo.S1FG) compared to untargeted delivery (unmodified AAV9.pCMV.S1FG) or control (unmodified AAV9.pCMV.lacZ); with cotreatment with CXCR4 antagonist AMD3100 abrogating this effect. Scale bars: 100 µm. Values represent the mean ± SEM. *n* = 4, 1‐way ANOVA with Dunnett post‐test.

Expression of an adhesion molecule, which serves to recruit streaming blood cells to the endothelium, is a tightly controlled privilege shared by microvascular endothelial cells,^[^ ^20^ ^]^ which cannot be replaced by subendothelial or parenchymal cells. Therefore, we tested the hypothesis that AAV9 encoding adhesion molecules would recruit leukocytes only when altered by endothelial retargeting modifications. In order to assess the ability of G2^CNN^ AAV9 to recruit leukocytes to an otherwise unstimulated microvascular endothelium, we constructed S1FG as a transgene, an artificial adhesion molecule consisting of the SDF1 (= stromal cell derived factor 1) chemokine head, a fractalkine stalk and a GPI (= glycosidolphosphatidylinositol)anchor (**Figure** **4**A).^[^ ^21^ ^]^ Using a cremaster model of intravital microscopy to analyze adherent leukocytes (Figure 4B,C), we found that the unmodified AAV9.pCMV.S1FG did not increase leukocyte adhesion compared to controls (AAV9.pCMV.LacZ). However, G2^CNN^ AAV9.pEndo.S1FG increased adherent leukocytes 4.6‐fold (Figure 4C,D). Of note, coapplication of AMD3100, which interrupts binding of SDF‐1 to its receptor CXCR4 on circulating leukocytes, entirely abrogated this effect (Figure 4D).

### 2.4 Prevention of Endothelial Activation in a Chronic Atherosclerosis Model

**Figure 5:**
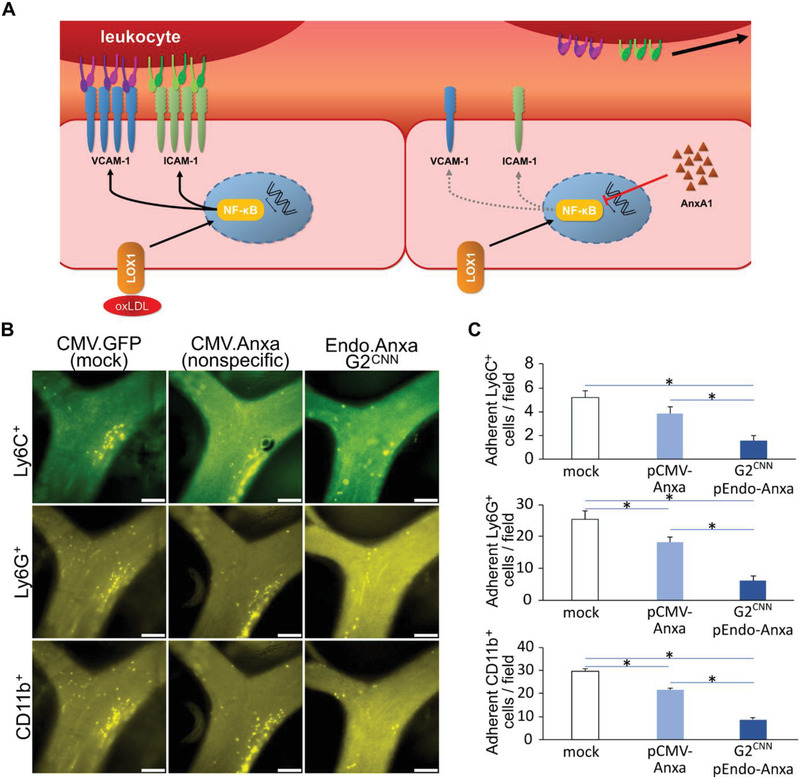
Endothelial retargeted of Annexin A1 abrogates leukocyte recruitment in chronic atherosclerotic mouse model. A) Endothelial activation and subsequent macrophage recruitment is a crucial part of atherosclerotic plaque formation and progression. B) Unmodified or endothelial retargeted Annexin A1 encoding AAV9 were tail vein injected to ApoE^−/‐^ mice on high fat diet. Ly6C^+^, Ly6G^+^, and CD11b^+^ leukocyte subpopulations were stained by intravenous injection of respective antibodies and visualized by intravital microscopy of carotid arteries. C) Adhesion of all three subtypes showed a significant decrease with endothelial retargeting (G2^CNN^ coated AAV9.pEndo.Anxa) in comparison to untargeted delivery (unmodified AAV9.pCMV.Anxa) or control (unmodified AAV9.pCMV.EGFP). Scale bars: 100 µm; *n* = 12, 1‐way ANOVA with Dunnett post‐test.

AAV vectors are known to have a long expression interval, at least in myocytic cells.^[^ ^8^ ^]^ To test whether this feature is capable pacifying a chronic inflammatory state such as atherosclerosis long‐term, we used a murine model of atherosclerosis, namely apoE^−/−^ mice fed with a high‐fat diet, which are characterized by aortic plaque formation (**Figure** **5**A). We utilized a G2^CNN^ AAV9.pEndo encoding the anti‐inflammatory protein Annexin A1 (Anxa1) ^[^ ^22^ ^]^ to prevent leukocyte recruitment to the endothelium of the aorta, a hallmark of plaque formation in hyperlipidemic mice. Upon systemic administration of G2CNNAAV9.pEndo‐Anxa1, we found a robust increase of Anxa1 transgene expression, when compared to uncoated AAV9.pEndo‐Anxa1 (Figure S4A, Supporting Information). 2 months after systemic application of 2.5 × 10^12^ vgs of G2^CNN^ AAV9, or wildtype AAV9 encoding Anxa1, carotid arteries were analyzed for leukocyte recruitment. We detected significantly less Ly6C^+^‐monocytes as well as Ly6G^+^‐neutrophils among the CD11b^+^‐leukocytes recruited to the carotid arterial wall of hyperlipidemic mice when G2^CNN^ AAV9.pEndo.Anxa1 was applied, compared to EGFP‐control vector or wildtype AAV9.pCMV.Anxa1 (Figure 5B,C). Bulk RNA sequencing on isolated aortic endothelial cells showed segregation cells from G2^CNN^ AAV9.pEndo.Anxa1 transduced animals into distinct clusters from those isolated from EGFP‐control vector transduced ones (Figure S4B,C, Supporting Information)

### 2.5 AAVs Affect Blood Pressure Control via Cas9‐Mediated Endothelial Nitric Oxide Synthase Excision

**Figure 6:**
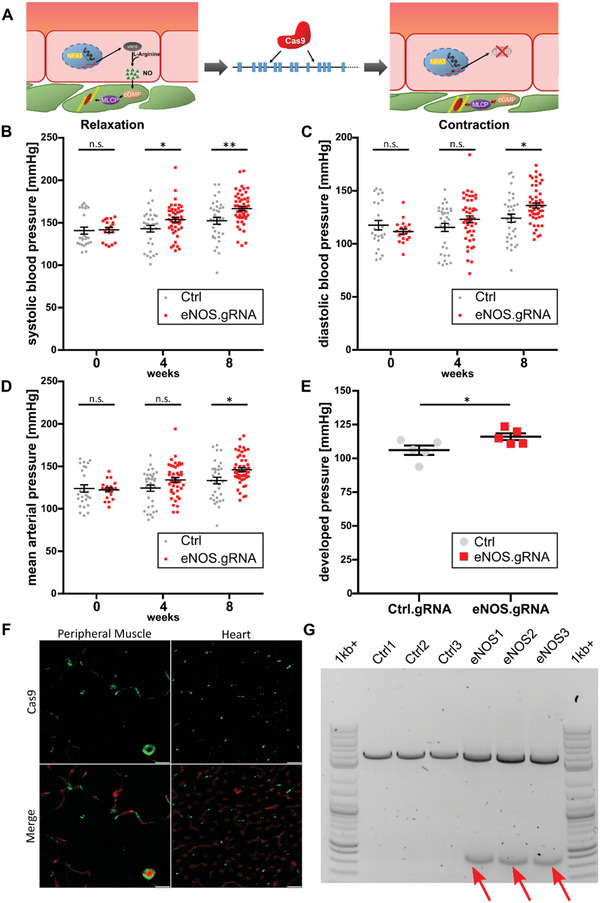
Blood pressure alteration by Cas9 mediated eNOS deletion with endothelial retargeted AAV9. A) Vasodilative effect of nitrous oxide synthesis by endothelial cells was targeted by deletion of exons 6 through 10 of the eNOS gene via CRISPR/Cas9, rendering overlaying smooth muscle cells incapable of relaxation. Conditional Cas9 knock‐in mice were transduced by unmodified AAV9.pEndo.Cre or G2^CNN^ coated AAV9.pEndo.Cre with sgRNA targeting eNOS. B–E) Noninvasive blood pressure measurements were taken at baseline, 4 and 8 weeks; along with cardiac catheterization at week 8. Systolic, diastolic, mean arterial, and developed pressure values were plotted (*n* = 6, 1‐way ANOVA with Dunnett post‐test). F) Cas9 expression (green) was observed in CD31^+^ cells (red) in both heart (left) and muscle (right) (Scale bars: 25 µm). G) Edited alleles (indicated by arrows) in magnetically sorted CD31^+^ cells of skeletal muscle were evident upon agarose gel separation (NEB 1kb+ ladder).

Whole body blood pressure control is—due to its vital function—a complex, multilayered, redundant phenomenon. Nevertheless, endothelial nitric oxide synthase (eNOS) plays a central role in vasodilation of microcirculatory arterioles by releasing the gaseous autacoid nitric oxide, which relaxes adjacent smooth muscle cell cytoskeletons (**Figure** **6**A), maintaining blood pressure at a physiological range.^[^ ^23^ ^]^ Here, we investigated in Cas9‐transgenic (Cas9‐tg) mice, whether a G2^CNN^ AAV9‐gRNA‐mediated knockout of endothelial NOS is capable of increasing systemic blood pressure (Figure 6A). We found that in Cas9‐tg mice,^[^ ^24^ ^]^ G2^CNN^ AAV9‐gRNAs designed to excise between exons 6 and 10 were sufficient to significantly increase mean arterial pressure by 23.7 ± 2.3 mmHg (vs 9.4 ± 4.7 mmHg in control animals) 2 months after systemic transduction in noninvasive measurements. Similar results were obtained for diastolic and systolic blood pressure (Figure 6B–D). At the 2 months timepoint, cardiac catheterization was performed, confirming the increase in blood pressure in the G2^CNN^ AAV9.gRNA^eN°S^ group compared to mock‐transduced controls (Figure 6E). In endothelial cells of these animals, where abundant Cas9 expression was observed (Figure 6F), editing of eNOS was obtained in 20.7% ± 1.6% of eNOS alleles in CD31^+^ endothelial cells (Figure 6G).

Besides heart and muscle endothelia, the G2^CNN^‐coated AAV9‐virus transduced a variety of tissue‐specific CD31^+^ endothelial cells, including aorta, liver, diaphragm, spleen, and brain (Figures S5–S7, Supporting Information). However, the amount of microvascular transduction of skeletal muscle and heart tissue was striking, since these tissues are within the tropism of the AAV9 serotype, raising the possibility that the original tropism might primarily steer the vector toward the endothelia of its target tissue. Concomitant muscle fiber transduction of G2^CNN^‐vectors indicates that feature. Moreover, analysis of fluorescently labeled vector capsids suggests entry via the abluminal plasmalemma, (Figure S8, Supporting Information), fitting the notion of an intact myotropism in place for G2^CNN^ AAV9, which only by virtue of EPs is attracted to neighboring endothelial cells.

## 3 Discussion

Here, we report retargeting of myotropic AAV9 virions by linking endothelial‐affine peptides to G2‐polyamidoamine dendrimers and fusing the latter to AAV9 capsids. Apparently, the 2 kDa PEG stabilizes the particle coating and at the same time enables slightly positive surface charge. PEG‐G2‐dendrimer coating alone doubled background endothelial transduction efficacy, whereas G2‐dendrimers armed with endothelial‐affine peptides EP^CNN^ or EP^SLR^ increased endothelial transduction four‐ or six‐fold, respectively. Consistently, the G2‐coating of AAV9 encoding for the artificial adhesion molecule S1FG provided leukocyte‐adhesion in microvessels only when coupled to the CNN‐peptide via a PEGylated linker. In chronic inflammatory processes such as atherosclerosis, endothelial‐retargeted AAV9‐transduction of anti‐inflammatory Anxa1 attenuates aortic recruitment of neutrophils ^[^ ^25^ ^]^ and monocytes in atherosclerotic plaques 2 months after transduction. In the same time interval, G2^CNN^ AAV9 encoding for eNOS‐gRNAs sufficed to increase systemic blood pressure in Cas9‐tg mice. Of note, we achieved transduction of all compartments of the arterial vessel tree, namely precapillary arterioles (cremaster model), large (carotid) arteries (atherosclerosis model) and resistance arteries (blood pressure model).

This finding was obtained while using the capsid of AAV serotype 9, which itself displays a comparatively high myotropism.^[^ ^26^ ^]^ Of note, G2‐ and peptide (EP^CNN^ or EP^SLR^) coating of AAV9 vectors did not alter this feature (Figure S1A,B,G, Supporting Information), nor the constitutive liver tropism of this serotype (Figure S6, Supporting Information). A permissive behavior of the G2^CNN^ or G2^SLR^‐ modified AAV9, transducing both, endothelial and myocytic cells in vivo, was abrogated by using an appropriate, endothelial‐specific promoter (pEndoglin). This in line with our observations using PAMAM for coating of adenovirus: the highest gain of transduction efficiency was achieved in CAR (Coxsackie‐and‐adenovirus receptor)‐negative cells, and the lowest one on low‐CAR cells,^[^ ^27^ ^]^ indicating that the G5‐coating of the Ad‐particle did not alter the wildtype tropism to its cognate receptor CAR. Hence, also here, we assume rather a “gain‐of‐function” and not an abrogation of intrinsic targeting ability of AAV9.

In contrast, complete detargeting of the AAV9 from the myocyte compartment by inserting the SLR motif directly in the virus capsid ^[^ ^13^ ^]^ abrogates myotropism, without enabling microvascular transduction of peripheral or cardiac endothelial cells. Of note, even when the AAV9^SLR^ strain is coated with PAMAM G2^SLR^, detargeting is still dominant and no endothelial transduction is detectable (Figure S9, Supporting Information). Taken together, endothelial retargeting of the AAV9 requires an intact vector capsid and an endothelial‐affine petide which has to be firmly attached to the capsid, e.g., by a PEGylated G2‐PAMAM. It is tempting to speculate whether these observations imply an abluminal transduction of the G2‐EP coated AAV9, given that it still traveling to and transducing myocytes (Figure S1F, Supporting Information), a feature we suppressed by promoter selectivity.

Of note, while with PEG‐G2 coating particles were colloidally stable, coating with unmodified G2‐PAMAM promoted partial aggregation and negatively interefered with the zeta potential measurement based on laser light scattering (data not shown). This is in contrast to PAMAM‐G5 coated adenovirus, where stable virus particle could be obtained.^[^ ^27^ ^]^ On the other hand, no overt toxicity was found when analyzing blood sera and samples of liver, spleen and heart of G2^CYS^‐AAV9 transduced pigs (Figure S2C and Tables S1 and S2, Supporting Information).

In conclusion, coating of AAV capsids with G2‐EPs achieves retargeting to endothelial cells. These results may open the door toward a broader AAV applicability in vascular disease models, e.g., plaque targeting in such as atherosclerotic mice, aortic targeting in mice with inherited vascular disease, e.g., Marfan syndrome or aortic aneurysm treatment. Moreover, endothelial transduction for investigations of microvascular growth and maturation may benefit from an endothelial retargeted AAV.

## 4 Experimental Section

### Virus Generation

The recombinant AAV9 (pseudotype rAAV2/9) was produced using the triple transfection method as described earlier.^[^ ^9^, ^28^ ^]^ Briefly, transfection of HEK293T cells (CRL‐3216, ATCC) was performed using one plasmid encoding for the transgene flanked by cis acting AAV2 inverted terminal repeats (ITR), a second plasmid providing AAV2 rep and AAV9 cap in trans, whereas a third plasmid (delta F6, Puresyn) supplemented adenoviral helper function using polyethylenimine (PEI‐Max, Polysciences Inc.). Titers of the AAVs were determined via quantitative realtime polymerase chain reaction (qRT‐PCR) against the ITR region.^[^ ^29^ ^]^

### Cell Culture and Peptide Selection

Human microvascular endothelial cells (HMEC‐1,CRL‐3243, ATCC) or HUVEC (Promocell) cells were cultured in 10% fetal calf serum (FCS) supplemented Dulbecco´s modified Eagle medium (DMEM) and Endothelial Cell Growth Medium 2 (Promocell), respectively. 2 × 10^11^ plaque forming units of a CX7C M13KE phage library (New England Biolabs) were incubated with 1 mL of 1% bovine serum albume (BSA)/DMEM in 1 × 10^7^ cells per tube of the HMEC‐1 or HUVEC cells and incubated for 120 min at 4 °C with gently rolling. After washing, bound phages were eluted with a 1 mL of 0.2 m Glycine‐HCl. 1 mL of selected phage eluate was incubated with 20 mL of an *E. coli* ER2738 bacteria suspension (A600 nm 0.5) (New England Biolabs,). Recovered phages were centrifuged at 4 °C/13 000 rpm/20 min. Then, the supernatant was transferred to a fresh tube with 1/6 volume of 20% PEG8000/2.5 m NaCl and incubated overnight at 4 °C. Thereafter, the phage was pelleted at 4 °C/13 000 rpm/15 min and the supernatant was discarded. The phage pellet was completely resuspended with 1 mL of TBS. 10 µL of phage elutes were incubated with 300 µL of *E. coli* ER2738. Each a single blue positive plaque was selected and amplified with 2 mL of an *E. coli* ER2738. Each selected phage DNA was amplified by direct PCR using KAPA HiFi DNA polymerase (Peqlab). Ten phage clone sequences were selected by sequencing analysis with the M13 phage primer sequences being: forward 5’‐ TTA TTC GCA ATT CCT TTA GTG G ‐3’, reverse 5’‐ CCC TCA TAG TTA GCG TAA CG ‐3’. Thereafter PCR products were separated and sequenced (Eurofins). Of seven varying sequences, CNNSGMRN was selected after alignment analysis by Clustal W program.

### Conjugate Syntheses

Amine terminated diaminobutane core PAMAM G2 dendrimers were purchased from Dendritic Nanotechnologies and Andrews ChemServices. Conjugation of a PAMAM G2 dendrimer, an NHS‐PEG‐OPSS linker, and endothelial cell specific transduction peptides (CNN or SLR) was performed, as described before.^[^ ^27^ ^]^ Briefly, 1 µmol of G2 was incubated with 4 µmol of NHS‐PEG_2kDa_‐OPSS (Rapp Polymere) dissolved in dimethyl sulfoxide (DMSO), loaded on a cation‐exchange column (Macro‐Prep High S; BioRad, Germany) and fractioned with a salt gradient from 0.6 to 3 m NaCl in 20 × 10^−3^ m HEPES (=4‐(2‐hydroxyethyl)‐1‐piperazineethanesulfonic acid), pH 7.4) solution. Thereafter, the product was filtrated by centrifuge filter devices (Amicon Ultra 3K, Merck) and the G2 content of the conjugate was determined by TNBS (= trinitrobenzene sulfonic acid) assay.

### Dendrimer Coating

Complexes of AAVs and G2 PAMAMs were formed by diluting indicated amounts of PAMAM dendrimers in Opti‐MEM I (Thermo Fisher). Viral particles were added to the diluted PAMAM solution, immediately mixed by gentle aspiration with the pipet tip and allowed to incubate for at room temperature for 30 min before further use.

### Zeta Potential

1 × 10^12^ vgs AAVs in 100 µL phosphate‐buffered saline (PBS) were mixed with 18 µg PAMAM_G2_‐PEG_2kDa_‐CYS conjugate (based on the weight of PAMAM dendrimer) in 100 µL in Hepes buffered glucose (HBG, 20 × 10^−3^ m HEPES, 5% w/v glucose, pH = 7.4), left for 30 min at 25 °C and then further diluted to 0.8 mL in a 1:1 mixture of PBS/HBG to be measured in the zetasizer Nano ZS (Malvern Pananalytical). Samples were run in clear disposable zeta cells at 25 °C with the dispersant selected as water (0.8872 cP viscosity), set to autovoltage (always reached 50.3 V) for a maximum of 100 runs twice with 60 s cool down time between aquisitions. Results were analyzed using Zetasizer Software v7.13 (Malvern) and only those extracted with good result quality as per the software.

### TEM

AAV were coated with PAMAM conjugate as described above and processed in principle as described by Chen.^[^ ^30^ ^]^ In brief, 2 µL sample was pipetted onto a formvar coated 300 mesh copper grid, left to evaporate for 6 h, then drop coated with 20 µL 1% phosphor tungstic acid solution for three minutes, excess liquid drained off and left to dry overnight. Samples were imaged on a Libra 120 system (Zeiss,) using a 120 kV operating filter and a LaB^6^ filament together with a Sharp:eye camera (TRS) and an in column camera Morada G2 (11 MP)

### Mouse Strains

Animal care and all experimental procedures were performed in accordance with the Animal Care and Use Committees of Bavaria (ROB‐55.2‐2532.Vet_02‐18‐99, ROB‐55.2‐2532.Vet_ 02‐21‐28, and ROB‐55.2‐2532.VET_02‐16‐137), Germany. Rosa26 mTmG (007576, The Jackson Laboratory) and Rosa26‐LSL‐Cas9 (024857, The Jackson Laboratory) mice were kindly provided by Ralf Adams (Max Planck Institute for molecular biomedicine, Muenster, Germany) and Roland Rad (Klinikum Rechts der Isar, Munich, Germany), respectively. ApoE^−/−^ mice provided by Oliver Söhnlein (Institute for Cardiovascular Prevention, Ludwig‐Maximilians‐University, Munich, Germany) were used in a C57Bl6 background and fed a high‐fat diet containing 21% fat and 0.15% cholesterol (ssniff, Soest) for 4 weeks. All AAV in vivo experiments were conducted with tail vein injection of 2.5 × 10^12^ vgs.

### AAV Infusion into mTmG Reporter Pigs

Transduction of mTmG reporter pigs were carried out as described before.^[^ ^31^ ^]^ Briefly, pigs (*n *= 2) were anesthetized, two 6F sheaths were inserted into the carotid artery and the external jugular vein, and left anterior descending artery was blocked by an over‐the‐wire balloon. AAV (1 × 10^14^ vgs) was injected through the lumen on this balloon while simultaneously blocking the anterior interventricular vein with a Swan Ganz catheter. Another load of virus (1 × 10^14^ vgs) was also injected intramuscularly into biceps femoris. The animals were sacrificed after 3 weeks, and the hearts were excised, sampled in a systematic manner,^[^ ^32^ ^]^ and snap frozen in isopentane for further investigation. Regions from the biceps femoris centered on the intramusclular injection site were also collected for flow cytometric analysis.

### Fluorescence Microscopy

Microscopy was performed with a Leica THUNDER Imager Tissue (Leica Microsystems,) equipped with a 63XxNA1.40 oil immersion objective. Optical zoom was utilized where applicable. For fluorescence excitation, SOLA light engine (Lumencor) was used. Acquisition was performed with 2048^2^ pixels. Image processing was performed using Leica LAS X software, with utilization of built‐in image deconvolution (small volume computational clearing).

The following antibodies were used for immunofluorescence staining: EGFP (CAB4211, Thermo Fisher), murine CD31 (BM4086, Origene), porcine CD31 (LCI‐4, Bio‐Rad), CRISPR‐Cas9 (7A9‐3A3, Novus Biologicals), AAV9 intact capsid (ADK9, Origene). Highly crossadsorbed Alexa Fluor conjugated secondary antibodies were used for fluorescent labeling (A‐11034, A48265, A‐11029, A‐11032, Thermo Fisher). GFP/CD31 double positive cells in immunofluorescence images were counted from ten sections with ImageJ.

### Flow Cytometry Analysis of Transduction

Tissue samples were digested as follows: 10 mg pieces of murine postischemic hearts were digested with Collagenase II (1 mg mL^−1^; C6885, Sigma) and DNase I (50 µg mL^−1^; D4263, Sigma) for 1 h. Porcine biceps femoris samples were sectioned into 50 mg pieces, digested first with 1 mg mL^−1^ papain (P3375, Sigma) for 30 min, followed by 1 mg mL^−1^ Collagenase/Dispase (Roche) for 30 min. Cell suspensions were pressed through a cell strainer (70 µm) and centrifuged at 300 × *g*. Red blood cells were lysed with an ammonium‐potassium buffer washed twice and stained for viability with trypan blue for murine and LIVE/DEAD (Thermo Fisher) for porcine cells. Murine cells were immediately stained with a specific antibody for CD31 (BM4086 , Origene) for 15 min, followed by secondary antibody staining (A48265, Thermo Fisher). Porcine cells were fixed with 4% w/v paraformaldehyde, permeabilized with Triton X100 and stained for EGFP (CAB4211, Thermo Fisher) and CD31 (LCI‐4, Bio‐Rad), followed by secondary antibody staining (A32731 and A48255, Thermo Fisher). Fluorescence measurements were carried out in an LSRFortessa (BD Biosciences).

### In Vivo Cremaster Microscopy

AAV9 S1FG injected C57Bl6 mice were anesthetized and the cremaster muscle exposed on a microscopic stage, as described.^[^ ^33^ ^]^ Morphometric parameters such as venular diameter, segment length, and leukocyte rolling velocity were assessed as described previously.^[^ ^34^ ^]^ The number of adherent cells per mm^2^ was determined using intravital microscopy (Olympus BX51WI microscope, water immersion objective ×20, 0.95 numerical aperture). All scenes were recorded using a CCD (=charge coupled device) camera (model CF8/1, Kappa) and virtual dub software for later off‐line analysis. During the entire observation, the cremaster muscle was superfused with thermocontrolled (35 °C) bicarbonate‐buffered saline. Postcapillary venules under observation ranged from 20 to 40 µm.

### Atherosclerosis Experiments

Eight weeks old Apoe^−/−^ mice were administered a nonspecifc AAV carrying Annexin A1 (2.5 × 10^12^ vgs), endothelial specific AAV carrying Annexin A1 (2.5 × 10^12^ vgs), or the vehicle (100 µL, NaCl) intravenously. The mice were fed a high‐fat diet containing 21% fat and 0.15% cholesterol (ssniff, Soest) for four weeks. All animal procedures were performed according to national guidelines for animal welfare and were approved by the Bavarian Animal Care and Use Committee (ROB‐55.2–2532.Vet_02–21–28).

### Intravital Microscopy of the Carotid Artery

Mice were placed in supine position and the right jugular vein was cannulated with a catheter for antibody injection. Intravital microscopy was performed after injection of a phycoerythrin (PE)‐conjugated antibody to Ly6G (1 µg, clone 1A8, 127608, BioLegend), an fluorescein (FITC)‐conjugated antibody to Ly6C (1 µg, HK1.1, 128022, BioLegend), and a PE‐conjugated antibody CD11b (1 µg, clone M1/70, 101208, BioLegend). Leukocyte‐endothelial interactions along the carotid artery were visualized using an Olympus BX51 microscope equipped with a Hamamatsu 9100‐02 electron multiplying CCD camera, and a 10 × saline‐immersion objective. Movies of 30 s were acquired and analyzed offline. One video per mouse was analyzed and video analysis was done in a blinded fashion.

### Fluorescence‐Activated Cell Sorting (FACS)

Aortas were digested with 1.25 mg mL^−1^ Liberase (Roche) and incubated for 1 h at 37 °C. The following antibodies were used for staining the single cell suspensions: CD45‐eFluor450 (30‐F11, 48‐0451‐82, Thermo Fisher), CD31‐APC (MEC13.3, 102510, BioLegend), Zombie Aqua Fixable Viability Kit (423102, BioLegend). Cells were washed with Hanks Balanced Salt Solution and directly sorted with a FACSAria III (BD) in extraction buffer from the PicoPure RNA‐Isolation kit (Thermo Fisher) and incubated for 30 min at 42 °C. RNA was extracted using the same kit according to manufacturers instructions.

### RNaseq Analysis

Library preparation for bulk‐sequencing of poly(A)‐RNA was done as described previously.^[^ ^35^ ^]^ Briefly, barcoded cDNA of each sample was generated with a Maxima RT polymerase (Thermo Fisher) using oligo‐dT primer containing barcodes, unique molecular identifiers (UMIs), and an adaptor. 5′‐Ends of the cDNAs were extended by a template switch oligo (TSO) and full‐length cDNA was amplified with primers binding to the TSO‐site and the adaptor. NEB UltraII FS kit (New England Biolabs) was used to fragment cDNA. After end repair and A‐tailing a TruSeq adapter was ligated and 3’‐end‐fragments were finally amplified using primers with Illumina P5 and P7 overhangs. In comparison to Parekh et al. (2016), the P5 and P7 sites were exchanged to allow sequencing of the cDNA in read1 and barcodes and UMIs in read2 to achieve a better cluster recognition. The library was sequenced on a NextSeq 500 (Illumina) with 63 cycles for the cDNA in read1 and 16 cycles for the barcodes and UMIs in read2. Data were processed using the published Drop‐seq pipeline (v1.0) to generate sample‐ and genewise UMI tables. Principal component analysis and heat map generation was carried out by the R package Pheatmap.

### Cas9 Mediated eNOS Deletion

Candidate single guide RNAs targeting murine eNOS were cloned into spCas9 plasmid (px459 #62988, Addgene) and evaluated by double strand break induction efficiency in Neuro2A cells (CCL‐131, ATCC) after polyethyleneimine transduction (PEI‐MAX, Polysciences Inc) by T7 endonuclease assay (New England Biolabs).^[^ ^36^ ^]^ Two sgRNAs, 5’‐GCG AGG GGA CCC CGC CAA CG‐3’ and 5“‐CAA TCC AGG CCC AAT CGG CA‐3”, were cloned into a self‐complementary AAV transgene plasmid along with pEndo.Cre cassette in the reverse orientation, and AAV9 virions were produced as described above (AAV9.pEndo.Cre.sgRNA^eNOS^).^[^ ^31^ ^]^ Adult LSL‐Cas9 mice (8 weeks of age) underwent initial noninvasive blood pressure measurements utilizing the CODA 2 noninvasive blood pressure measurement system (Kent Scientific).^[^ ^37^ ^]^ After baseline blood pressure measurement, 2.5 × 10^12^ vgs of either uncoated AAV9.pEndo.Cre or G2^CNN^ coated AAV9.pEndo.Cre.sgRNA^eNOS^ was injected via tail vein injection. Noninvasive blood pressure measurements were repeated after 4 and 8 weeks. 8 weeks after AAV‐injection, mice underwent left‐ventricular pressure volume loop recordings. To this end, mice were anaesthetized by intraperitoneal injection of MMF (**M**idazolam 5 mg kg^−1^ bodyweight, **M**edetomidin 0.5 mg kg^−1^ bodyweight and **F**entanyl 0.05 mg kg^−1^ bodyweight). After complete sedation a pressure‐volume catheter (Scisense P/V Catheter, 1.2F, 3.5 mm electrode spacing, Transonic) was inserted into the left ventricle via the right carotid artery. After stabilization of the blood pressure, 30 P/V loops were analyzed using the LabChart 8 Software. Heart and gastrocnemius muscles were flash frozen in isopentane.

Cryo‐sections were stained with CRISPR‐Cas9 antibody (7A9–3A3, Novus Biologicals) and CD31 antibody (BM4086 , Origen). DNA extraction was carried out by column purification (Nucleospin Tissue XS, Macherey‐Nagel). PCR of the edited region was carried out by Q5 polymerase (New England Biolabs) using the primers 5′ GAG GCA ATC TTC GGT GAG TGA CCC T 3′ and 5′ AAG GGG AGG AGC ATG GAA GAA AGC A 3′. Ratio of excised to wildtype amplicons were determined by quantifying band intensities on agarose gel by ImageJ.

### Statistical Analysis

Data were presented as Mean ± SEM (standard error of the mean). Unpaired, 2‐tailed Student's *T*‐test was used to compare means of two groups, whereas, 1‐way ANOVA (analysis of variance) with Dunnett post‐test was applied for comparison of multiple groups. Statistical significance was defined as *p<0.05*. Stars denote * = p<0.05; ** p<0.01,*** p<0.001,**** p<0.0001. Statistical analysis and graph generation was performed with Graphpad 8.0.1.

## Conflict of Interest

A patent was filed by S.L., T.B., and C.K.

## Supporting information

Supporting Information

## Data Availability

The data that support the findings of this study are available from the corresponding author upon reasonable request.
