# Supplementary material for: Endothelial Retargeting of AAV9 In Vivo
Source: Adv Sci (Weinh). 2022 Jan 12;9(7):2103867. doi: 10.1002/advs.202103867 (PMC8895123; doi:10.1002/advs.202103867)
Supplement: Supplementary file 1 — Supporting Information [file ADVS-9-2103867-s001.pdf]

ADVANCED SCIENCE
Open Access

Supporting Information

for *Adv. Sci.,* DOI: 10.1002/advs.202103867

Endothelial Retargeting of AAV9 In Vivo

Tarik Bozoglu§, Seungmin Lee§, Tilman Ziegler§, Victoria Jurisch, Sanne Maas, Andrea Bähr, Rabea Hinkel, Amelie Hoenig, Anjana Hariharan, Simon Decker, Haider Sami, Tobias Koppara, Ruppert Oellinger, Oliver J. Müller, Derk Frank, Remco Megens, Peter Nelson, Christian Weber, Angelika Schnieke, Markus Sperandio, Gianluca Santamaria, Roland Rad, Alessandra Moretti, Karl-Ludwig Laugwitz, Oliver Soehnlein, Manfred Ogris, Christian Kupatt#

Supporting Information

Endothelial Retargeting of AAV9 In Vivo

Tarik Bozoglu[1,2,§], Seungmin Lee[1,2,§], Tilman Ziegler[1,2,§], Victoria Jurisch[1,2], Sanne Maas[2,6], Andrea Bähr[1,2], Rabea Hinkel[1,2,3], Amelie Hoenig[1,2], Anjana Hariharan[1,2], Simon Decker[4], Haider Sami[4], Tobias Koppara[1,2], Ruppert Oellinger[10], Oliver J. Müller[5], Derk Frank[5], Remco Megens[6], Peter Nelson[7], Christian Weber[2,6], Angelika Schnieke[8], Markus Sperandio[9], Gianluca Santamaria[1,2], Roland Rad[10], Alessandra Moretti[1,2], Karl-Ludwig Laugwitz[1,2], Oliver Soehnlein[2,6,11], Manfred Ogris[4], Christian Kupatt[1,2#]

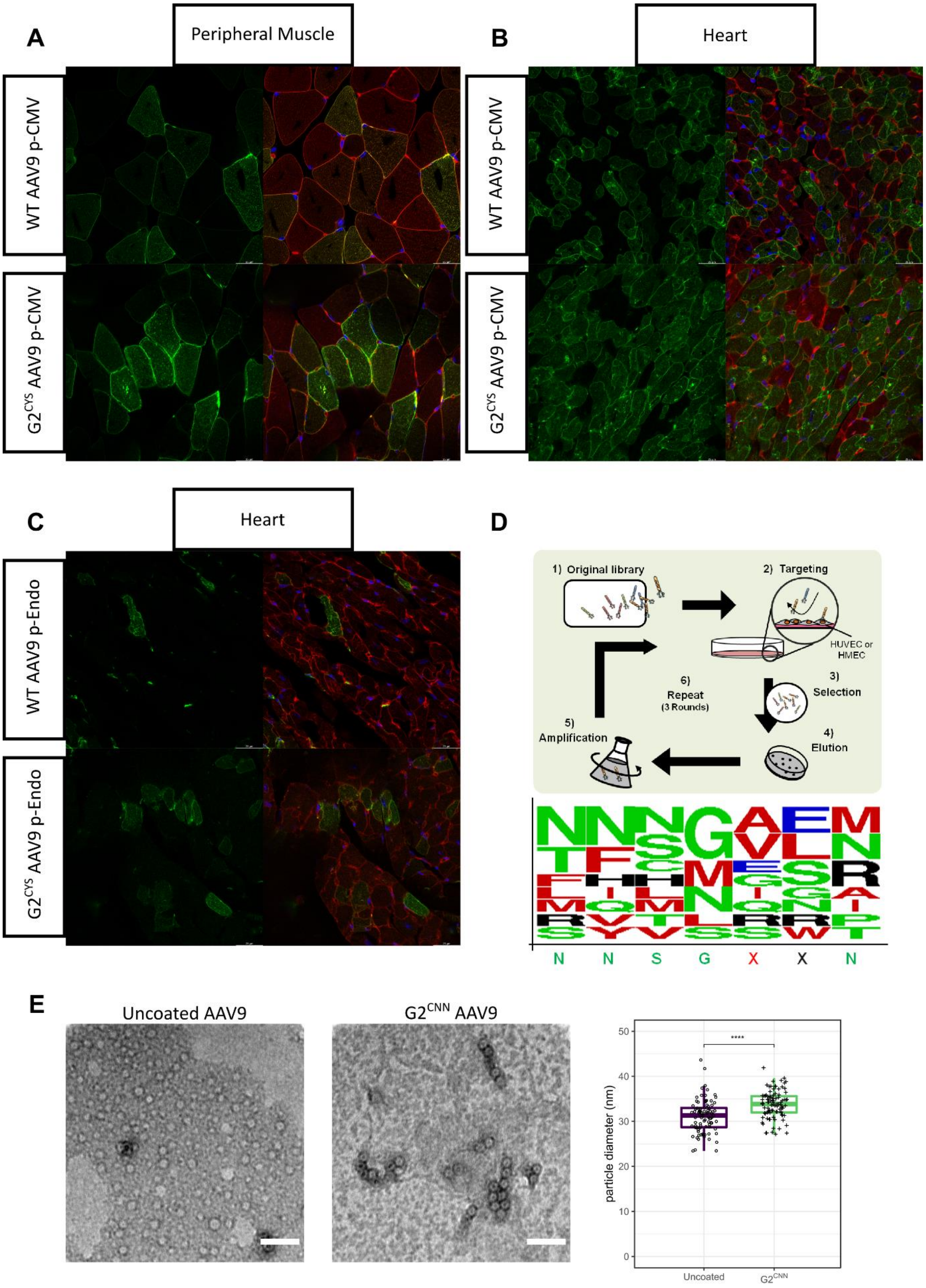
A
Peripheral Muscle
WT AAV9 p-CMV
G2CYS AAV9 p-CMV
B
Heart
WT AAV9 p-CMV
G2CYS AAV9 p-CMV
C
Heart
WT AAV9 p-Endo
G2CYS AAV9 p-Endo
D
1) Original library
2) Targeting
HUVEC or HMEC
3) Selection
4) Elution
5) Amplification
6) Repeat (3 Rounds)
N N S G X X N
E
Uncoated AAV9
G2CNN AAV9
particle diameter (nm)
Uncoated
G2CNN
****

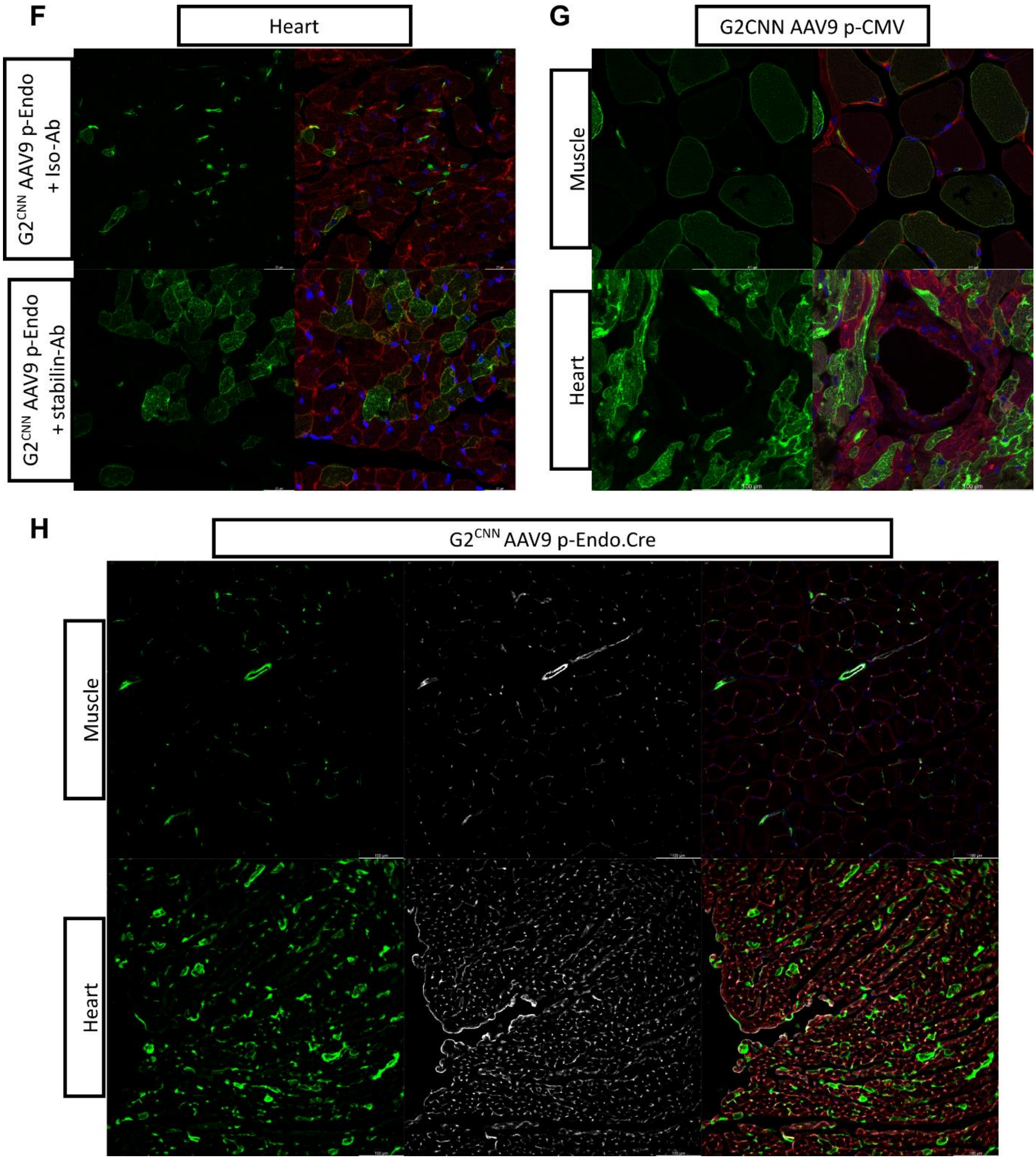

Figure S1: Influence of G2 coating on in vivo AAV9-Cre transduction of mTmG mice. **A** Peripheral muscle transduction of mTmG mice with unmodified (upper row) and $G2^{CYS}$ coated (lower row) AAV9.pCMV.Cre. **B** Heart transduction of mTmG mice with unmodified (upper row) and $G2^{CYS}$ coated (lower row) AAV9.pCMV.Cre. **C** Heart transduction of mTmG mice with unmodified (upper row) and $G2^{CYS}$ coated (lower row) AAV9.pEndo.Cre. **D** Biopanning procedure with CX7C M13KE phage display library and the resulting consensus peptide sequence. **E** Representative transmission electron micrograph of AAV9 and AAV9 coated with $G2^{CNN}$ (Scale bars: 100nm); along with comparison of particle diameters). **F** mTmG mouse heart transduction with $G2^{CNN}$ coated AAV9.pEndo.Cre 30 minutes after pre-injection with isotype control IgG_k (upper row) or anti-*Stab2* antibody (lower row). **G** Muscle and heart transduction of mTmG mice with $G2^{CNN}$ coated AAV9.pCMV.Cre. (Scale bars: 25µm; green: GFP, red: dtTomato, blue: DAPI). **H** Costaining of CD31 (white) with eGFP and dTomato in heart and muscle of mice transduced with $G2^{CNN}$ coated AAV9.pEndo-Cre.

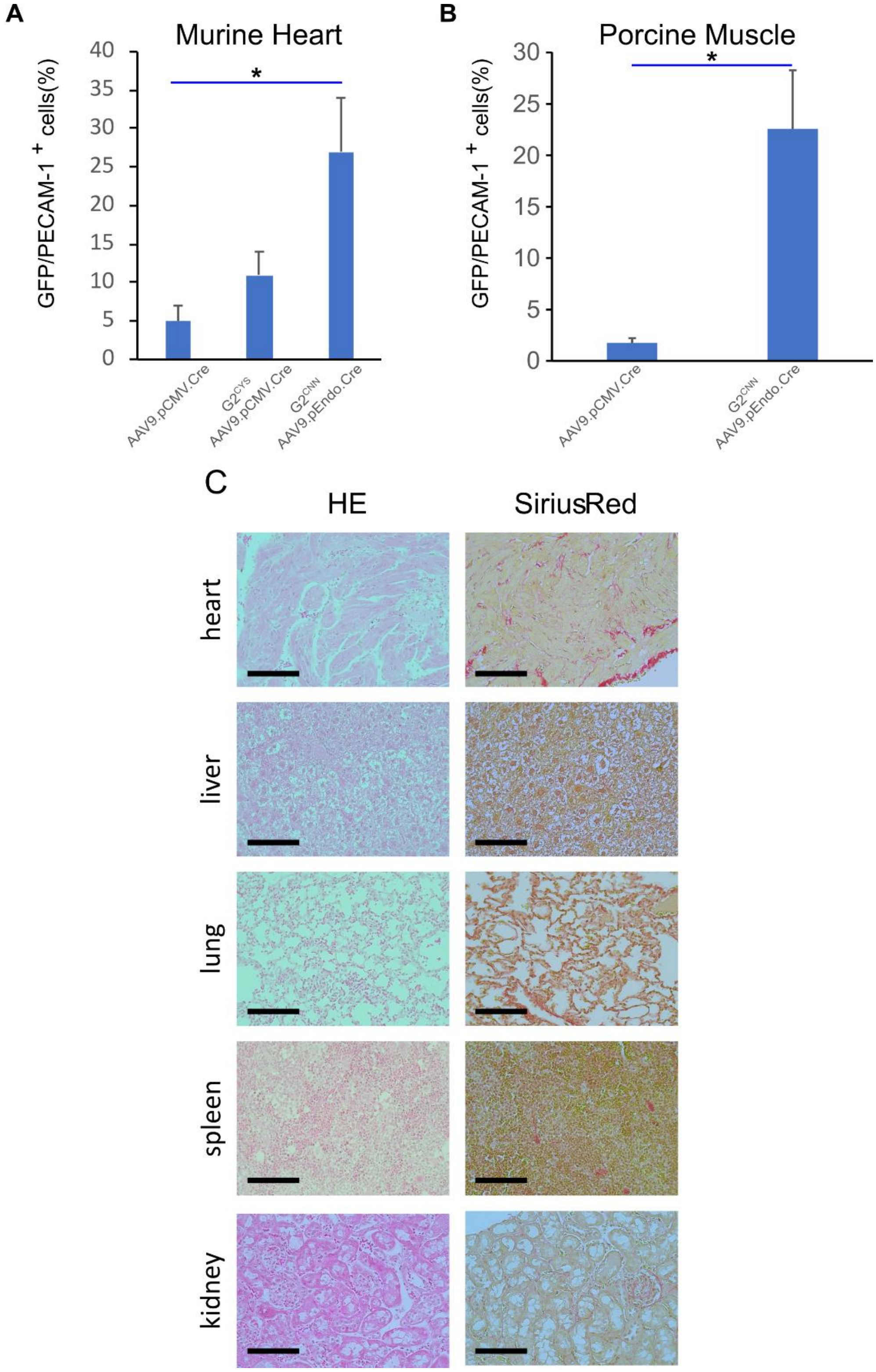

Figure S2: Quantification of endothelial transduction

A Percentage of GFP expressing CD31^{+} cells in mTmG mouse hearts transduced with AAV9.pCMV.Cre, G2 coated AAV9 pCMV.Cre or G2^{CNN} coated AAV9.pEndo.Cre. (n=3, p<0.05, Student's T test) **B** Percentage of GFP expressing CD31^{+} cells in mTmG hindleg muscle transduced with AAV9.pCMV.Cre or G2^{CNN} coated AAV9.pEndo.Cre. (n=5, p<0.05, Student's T test) **C** Hematoxilin-eosin staining of heart, liver, lung, spleen and kidney shows

no apparrent pathologies. Furthermore, Sirius Red staining shows no increase in organ fibrosis (scale bar 100µm).

	Ctrl	$G2^{CNN}$AAV9pEndo	p-value
Urea [mg/dl]	26.1 ± 0.66	26.77 ± 0.79	0.62
AlkPhos [U/l]	49.57 ± 3.77	48.77 ± 5.04	0.36
GPT [U/l]	51.8 ± 2.8	52.0 ± 3.77	0.91
gammaGT [U/l]	9.43 ± 0.47	8.87 ± 0.91	0.67

Supplementary Table 1: Clinical chemistry results demonstrate no change in liver and kidney function upon $G2^{CNN}$ coated AAV9 pEndo.Cre transduction

	Ctrl	$G2^{CNN}$AAV9pEndo	p-value
WBC [10^3/µl]	1.49 ± 0.21	1.38 ± 0.10	0.73
RBC [10^6/µl]	8.79 ± 0.40	8.47 ± 0.07	0.55
Hct [%]	49.40 ± 1.81	48.33 ± 0.81	0.68
Hb [g/dl]	13.17 ± 0.52	12.47 ± 0.10	0.34
MCV [fl]	56.27 ± 0.78	57.07 ± 0.55	0.53
MCH [pg]	15.00 ± 0.16	14.73 ± 0.05	0.27
MCHC [g/dl]	26.67 ± 0.47	25.80 ± 0.24	0.25
Plt [10^3/µl]	45.00 ± 6.24	42.67 ± 3.66	0.81

Supplementary Table 2: Full blood count shows no difference between $G2^{CNN}$ coated AAV9 pEndo.Cre transduced animals compared to control animals

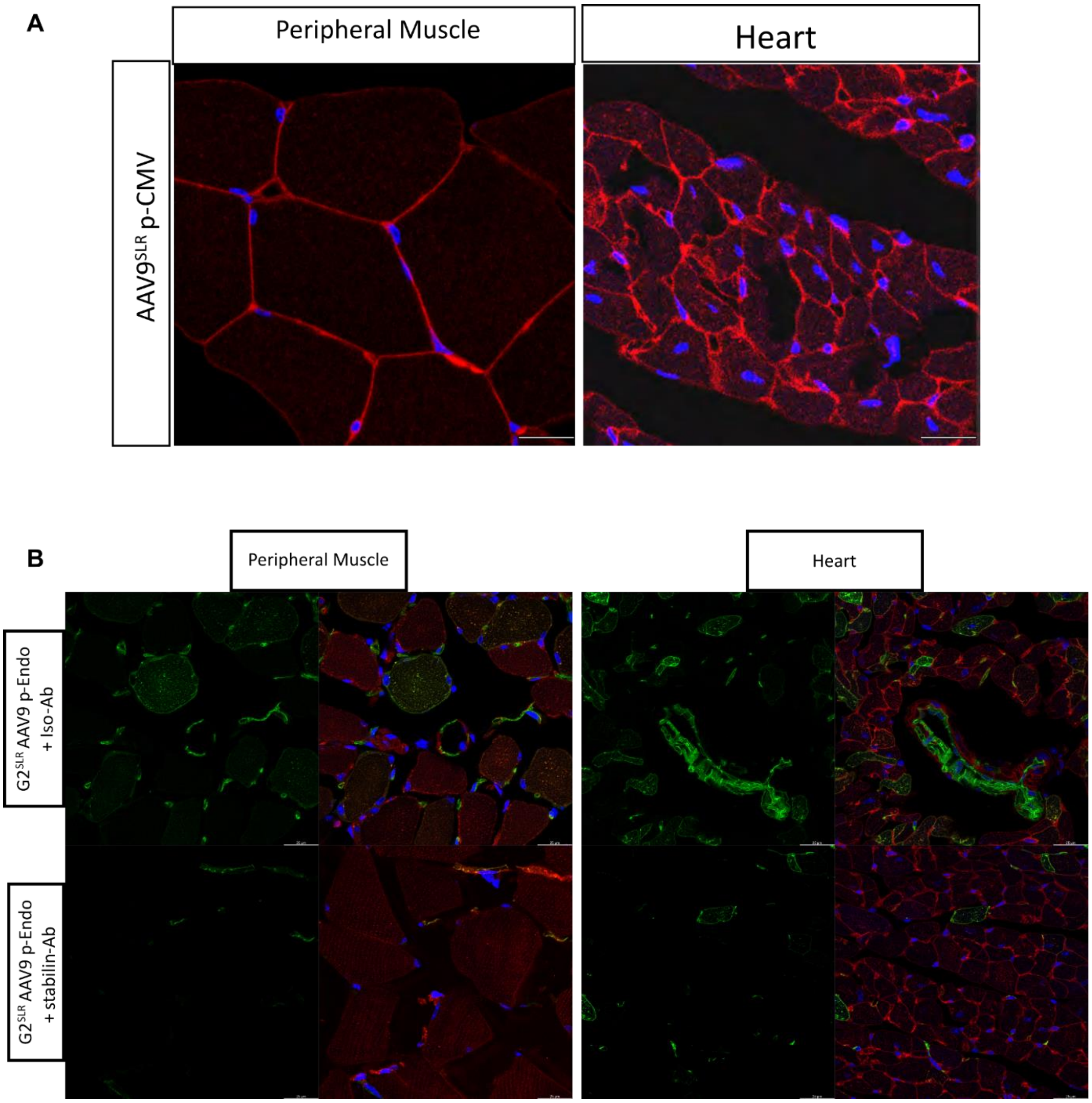

Figure S3: SLRSPPS peptide display on viral coat and on dendrimers.
A mTmG mouse peripheral muscle (left) and heart (right) transduction with AAV9^{SLR}.pCMV.Cre. **B** mTmG mouse peripheral muscle (left) and heart (right) transduction with G2^{SLR} coated AAV9.pEndo.Cre 30 minutes after pre-injection with isotype control IgG$_{k}$ (upper row) or anti-*FGFR3* antibody (lower row). (Scale bars: 25µm; green: GFP, red: dtTomato, blue: DAPI).

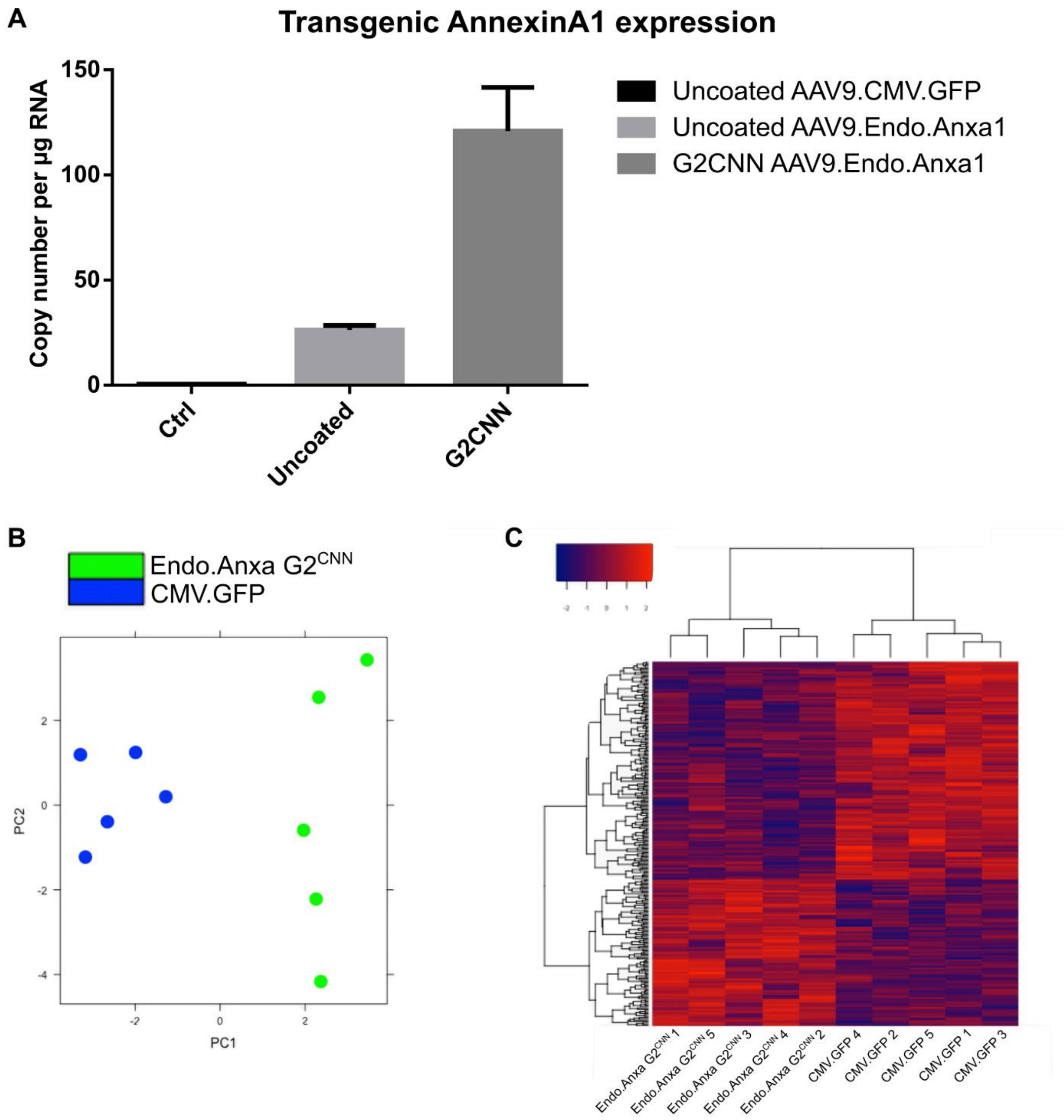

Figure S4: Bulk RNA sequencing

A ApoE$^{-/-}$ mice on high fat diet were transduced with either AAV9.pCMV.eGFP or with G2^{CNN} coated AAV9.pEndo.Anxa1. Transgenic AnnexinA1 expression in magnetically sorted skeletal muscle CD31^{+} cells. **B, C** Aortic endothelial cells were FACS sorted and bulk RNA sequenced**.** Principal component analysis was performed on the RNAseq data (**B**) and a Heat Map (**C**) of differentially regulated genes was constructed. (n=5).

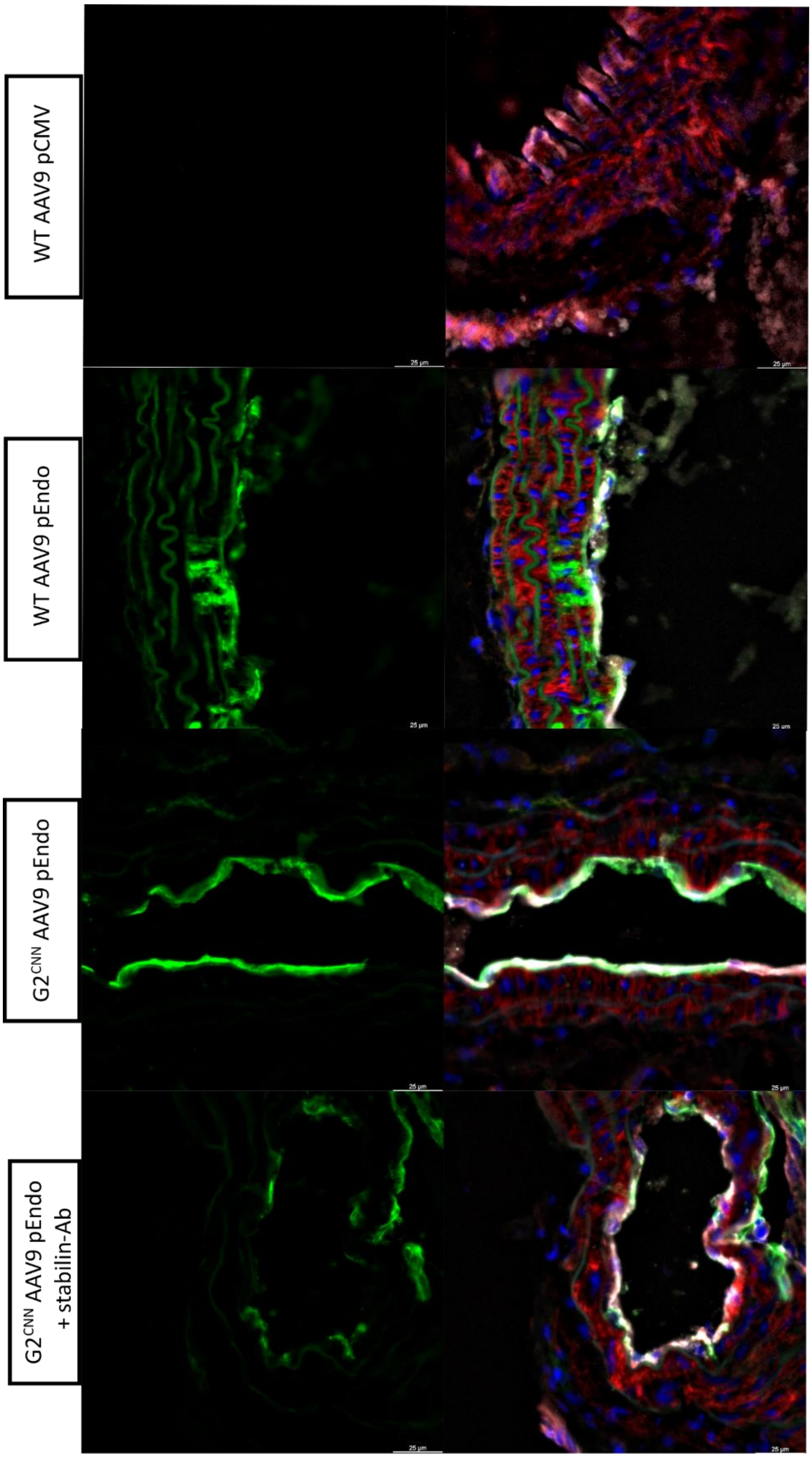

Figure S5: Aortic transduction

mTmG mouse aorta transduction with unmodified AAV9.pCMV.Cre (first row), unmodified AAV9.pEndo.Cre (second row), G2^{CNN} coated AAV9.pEndo.Cre (third row) and G2^{CNN} coated AAV9.p-Endo.Cre 30 min after injection of anti-*Stab2* antibody (fourth row). (Scale bars: 25µm; green: GFP, red: dtTomato, white: CD31, blue: DAPI).

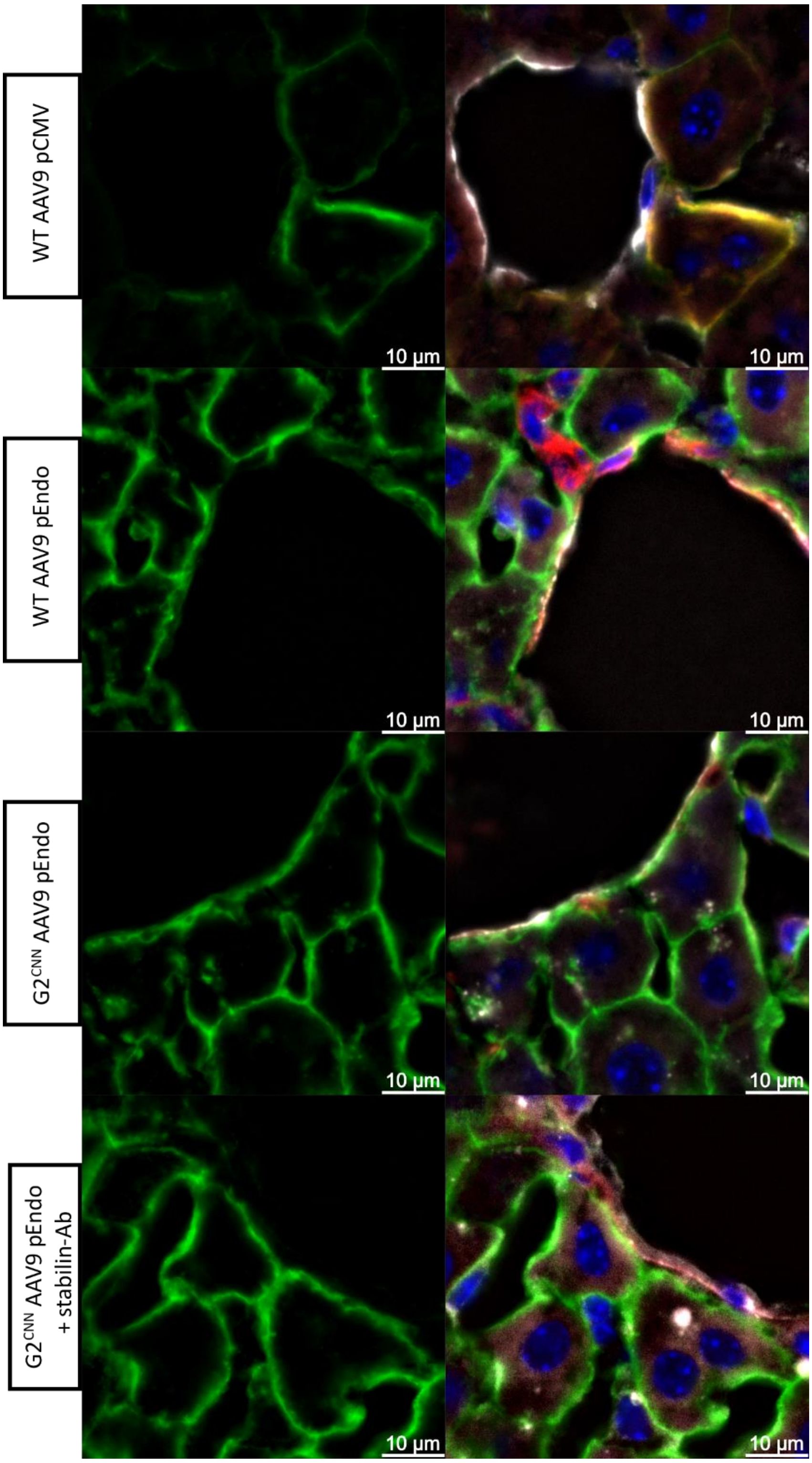

Figure S6: Liver transduction
mTmG mouse liver transduction with unmodified AAV9.pCMV.Cre (first row), unmodified AAV9.pEndo.Cre (second row), G2^{CNN} coated AAV9.pEndo.Cre (third row) and G2^{CNN} coated AAV9.p-Endo.Cre 30 min after injection of anti-*Stab2* antibody (fourth row). (Scale bars: 10µm; green: GFP, red: dtTomato, white: CD31, blue: DAPI).

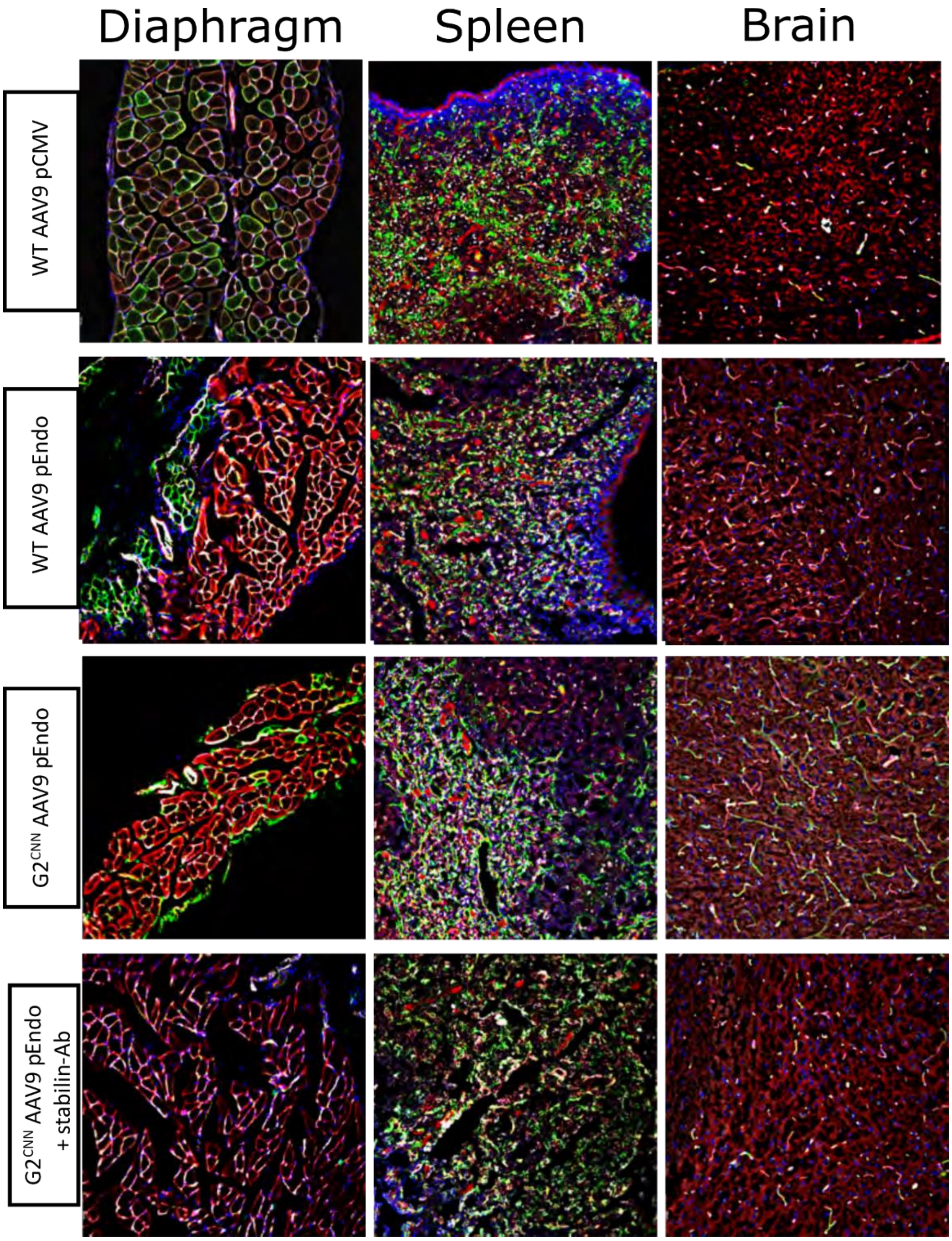

Figure S7: Transduction of diaphragm, spleen,and brain.
Transduction of the diaphragm (left), spleen (mid) and brain (right) tissues of mTmG mice with unmodified AAV9.pCMV.Cre (first row), unmodified AAV9.pEndo.Cre (second row), G2^{CNN} coated AAV9.pEndo.Cre (third row) and G2^{CNN} coated AAV9.p-Endo.Cre 30 min after injection of anti-*Stab2* antibody (fourth row). (Scale bars: 25µm; green: GFP, red: dtTomato, white: CD31, blue: DAPI).

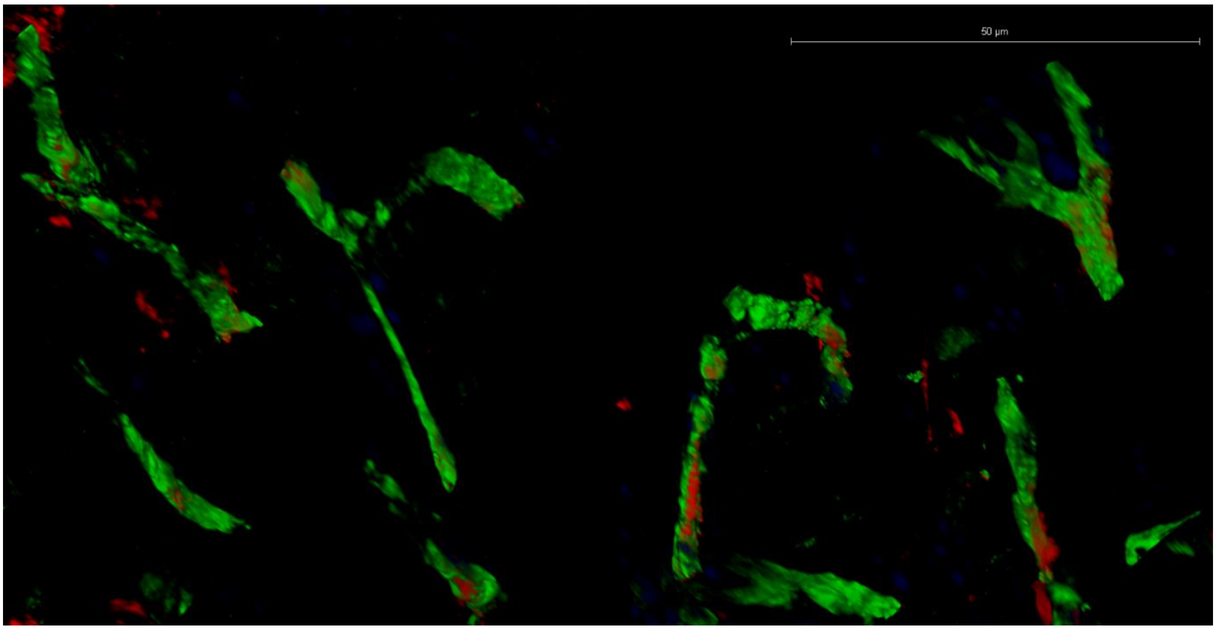

Figure S8: Abluminal Entry.
Muscle capillaries of wildtype mouse injected with G2^{CNN} coated AAV9.pCMV.LacZ, and sacrificed and perfusion fixed 1 hour after injection. 50µm sections were stained and imaged, 3D image reconstructed from Z-stacks. Scale bar: 50µm, green: CD31, red: AAV9, blue:DAPI.

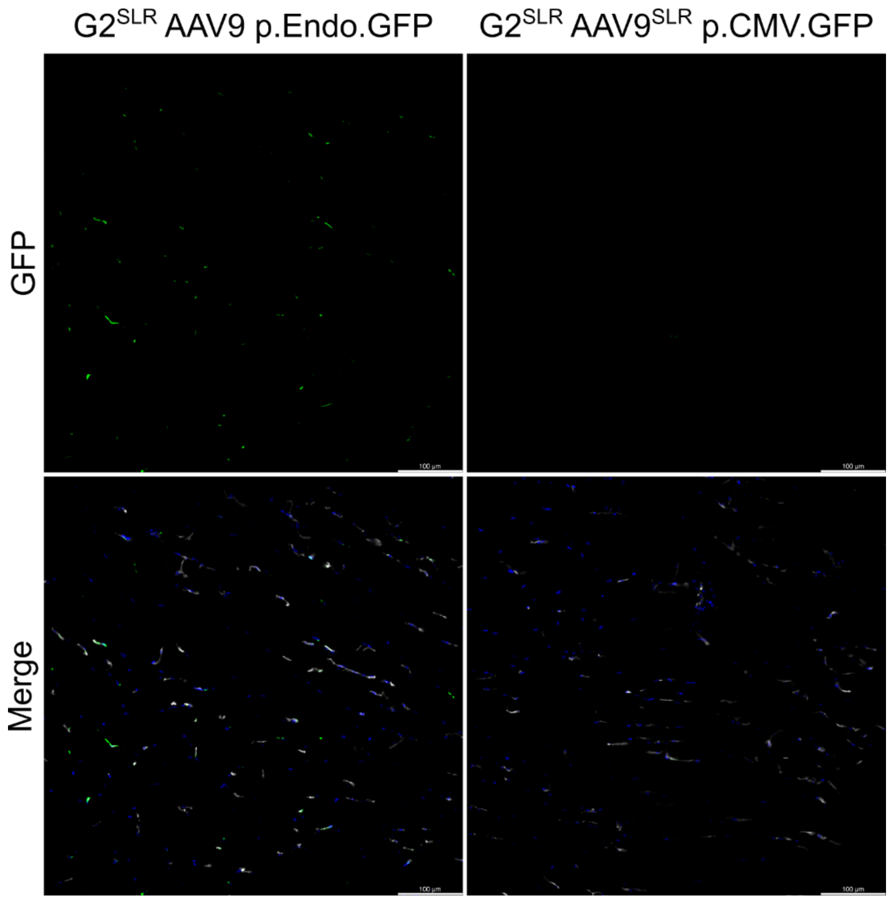

Figure S9: G2^{SLR} coated AAV9^{SLR}.

Muscle sections from wildtype mice transduced with G2^{SLR} coated AAV9.pEndo.EGFP (left) or G2^{SLR} coated pAAV9^{SLR}.pCMV.EGFP (right). Scale bars: 100µm; green: EGFP, white: CD31, blue: DAPI
